# Physical Therapy Perspectives for Medial Tibial Stress Syndrome in a Novice Runner: A Case Report

**DOI:** 10.7759/cureus.67647

**Published:** 2024-08-23

**Authors:** Swapnil U Ramteke, Pratik R Jaiswal

**Affiliations:** 1 Sports Physiotherapy, Ravi Nair Physiotherapy College, Datta Meghe Institute of Higher Education & Research, Wardha, IND

**Keywords:** kinesio taping, overuse injury, runners, novice, medial tibial stress syndrome

## Abstract

Medial tibial stress syndrome (MTSS), commonly referred to as shin splints, is a prevalent overuse injury observed in runners, particularly those who are inexperienced. This condition is marked by pain along the distal anteromedial region of the tibia, often resulting from repetitive stress and insufficient adaptation of the musculoskeletal system. This case report examines the physical therapy strategies utilized in the evaluation and management of MTSS in a novice runner. The subject of the study was a 22-year-old male novice runner who reported severe pain along the medial side of the tibia, which intensified during running activities. The physical therapy approach adopted was multifaceted, incorporating initial pain relief, relative rest, and a focus on strengthening and enhancing flexibility in the lower extremities, alongside proprioceptive training and mobilization techniques. A significant emphasis was placed on educating the patient. The patient participated in a progressive loading program designed to facilitate tissue healing and reduce the likelihood of recurrence. Over a span of six weeks, the patient exhibited considerable improvement in both symptoms and pain levels. Functional evaluations revealed increased strength and flexibility in the lower extremities. This case report underscores the effectiveness of comprehensive physical therapy interventions in the management of MTSS among novice runners. Thorough clinical assessments and customized rehabilitation programs were essential in addressing underlying issues and fostering recovery. These results highlight the necessity of personalized rehabilitation strategies to optimize treatment outcomes for individuals suffering from MTSS.

## Introduction

Medial tibial stress syndrome (MTSS) is a prevalent overuse injury that impacts the lower extremities, especially among athletes and military personnel. This syndrome is characterized by pain along the anteromedial aspect of the tibia brought on by exercise, and it signifies an initial phase in the progression of tibial stress fractures [1]. MTSS presents as an overuse injury marked by excessive stress on the tibial bone and accompanying periostitis. This condition is commonly observed in people participating in activities that involve repetitive impact, like running, jumping sports, and military training [2]. The prevalence of MTSS varies from 13.6% to 20% in runners and may escalate to 35% in military recruits. Factors like substantial rises in training intensity, workout duration, and activities with high impact can make individuals more susceptible to MTSS and consequent bone stress injuries. Studies on military recruits going through basic training have additionally found a link between vitamin D deficiency and an increased likelihood of stress injuries [3]. MTSS is a common reason for exercise-induced pain, characterized by pain along the inner back part of the shin, which is felt during exercise and when touched, covering an area of over 5 cm [4]. MTSS is probably caused by excessive strain on the medial tibia, with inflammation of the periosteum potentially contributing to the condition. This phenomenon arises from the persistent traction exerted by tendons on the periosteum of the tibia, a situation known as traction periostitis. Periostitis refers to a medical condition characterized by the inflammation of the periosteum, which is the connective tissue layer enveloping the bone. This condition is typically chronic and must be distinguished from stress fractures. Symptoms include tenderness, swelling of the affected bone, and persistent aching pain. When the muscle attachment exerts a pulling force on this layer, it is specifically termed traction periostitis [2].

MTSS has the potential to progress to a tibial stress fracture, as microtrauma to the cortical bone can lead to a complete fracture. Nevertheless, not all people with MTSS will experience a tibial stress fracture [5]. The typical clinical manifestations of MTSS consist of pain along the medial edge of the tibia, increased discomfort during physical activity, and acute pain when palpated [6]. The exact pathophysiology of shin splints remains not completely comprehended. The widely acknowledged hypotheses involve the theory of fascial traction and the theory of tibial bone overload. As per the fascial traction theory, shin splints are mainly triggered by the inflammation of the periosteum caused by excessive traction. This ailment is thought to be associated with inflammation arising from the overburden of muscles, tendons, and the periosteum encircling the tibia, frequently worsened by repetitive training [7]. The theory of tibial bone overload proposes that repeated overloading of the tibia causes micro-injuries. This persistent overloading leads to abnormal tibial loading, which then results in changes in biomechanics and musculoskeletal pain, typical of shin splints [8]. Furthermore, the diagnosis of shin splints generally requires an assessment of the patient's medical history, a thorough physical examination, and the use of medical imaging methods such as computed tomography. Various risk factors are associated with the onset of shin splints, which include intrinsic factors like being female, body mass index, navicular drop, and irregular gait patterns. Additionally, extrinsic factors, for instance, extended periods of walking or running, also contribute to this condition [9]. During the stance phase of running, the maximum eversion moment of the ankle was found to be greater in individuals with MTSS compared to those without the condition. Notably, even after the symptoms of MTSS have resolved, the running biomechanics of individuals with a history of MTSS continue to differ from those of individuals who have never experienced the condition [10].

Kinesiology taping (KT) is employed in the rehabilitation of athletic injuries due to its properties. These characteristics can help to increase the space between tissues, which in turn can promote blood circulation and lymphatic drainage. KT is recognized for its ability to enhance muscle flexibility, provide effective musculoskeletal support, and improve motor performance. Additionally, it can help decrease discomfort and enhance neuromuscular facilitation through the stimulation of skin mechanoreceptors [11]. In the acute phase, patients must practice "relative" rest and avoid engaging in sports activities, depending on the severity of their symptoms. Cryotherapy is commonly utilized during this initial stage. Treatment strategies include addressing dysfunctions, implementing proprioceptive training, and utilizing splints and orthotics, along with stretching and strengthening exercises. A daily regimen of calf stretching and eccentric calf exercises is advised to alleviate muscle fatigue. Furthermore, additional exercises should focus on enhancing the strength of the tibialis anterior and other muscles that facilitate foot inversion and eversion [12].

## Case presentation

The sports physical therapy department received a 22-year-old male novice runner complaining of pain in the bilateral anteromedial region of the tibia. He has been running sporadically for the past six months, around four to five days a week for 30-45 minutes each day. The pain started gradually after running approximately 1 km on a concrete surface. Despite resting for four days after the initial pain, it recurred after running 700 meters. The patient consulted a local orthopedician who advised rest and analgesics, but the patient did not follow the prescription and continued running until the pain resurfaced. Eventually, he stopped running altogether after two to three weeks and sought medical advice again. The orthopedician recommended radiographic evaluation to rule out any bony pathology, which showed no signs of fractures. Subsequently, the patient was referred to sports rehabilitation.

Clinical assessment

The patient specified a pain level of 7/10 on activity on the numerical pain rating scale during the physical examination and also exhibited grade 3 tenderness. Additionally, bilateral tightness in the ankle plantarflexors was observed. The results of manual muscle testing can be found in Table 1, while the active range of motion of the ankle before and after treatment is detailed in Table 2. Furthermore, the patient reported a pain level of 8/10 while walking over the anteromedial tibia bilaterally on the numerical pain rating scale.

**Table 1:** Bilateral manual muscle testing.

Muscle group	Pre-treatment	Post-treatment
Ankle dorsiflexors	4-/5	5/5
Ankle plantar flexors	5/5	5/5
Ankle invertors	4-/5	5/5
Ankle evertors	4-/5	5/5

**Table 2:** Bilateral ankle range of motion.

Movement	Pre-treatment	Post-treatment
Ankle dorsiflexion	0-10	0-20
Ankle plantarflexion	0-45	0-50
Ankle inversion	0-25	0-35
Ankle eversion	0-10	0-20

Therapeutic intervention

**Figure 1:**
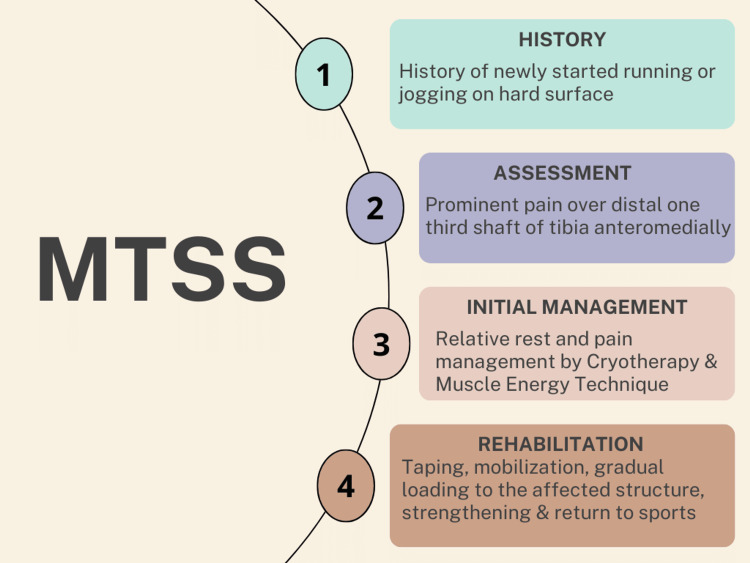
Timeline and proceeding during the rehabilitation. MTSS: medial tibial stress syndrome

**Figure 2:**
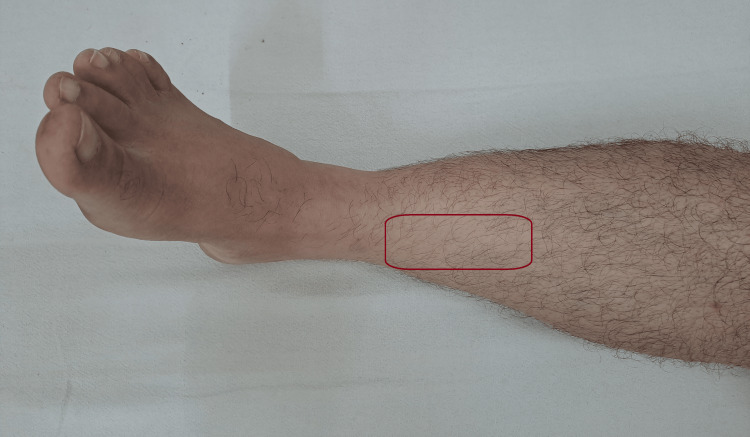
Area of tenderness.

**Figure 3:**
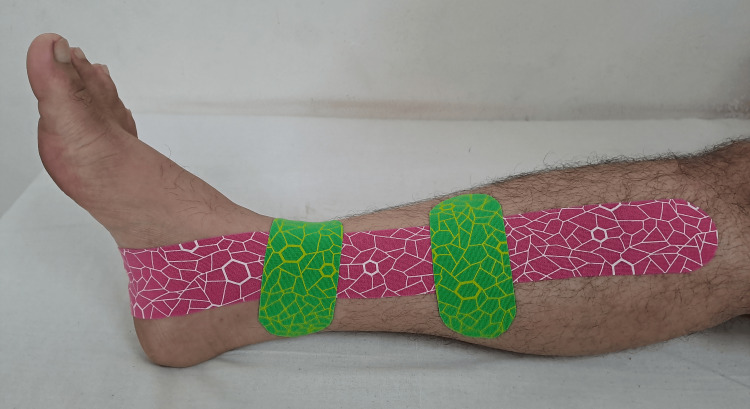
The Kinesio taping technique involves the application of an I strip (pink) that is stretched to 25% of its original length. One end of the tape is anchored at the anteromedial aspect of the proximal tibia, while the other end is positioned below the navicular bone without any stretch. Additionally, two blocks (green) are applied with a 25% stretch in the center, accompanied by two bases placed alongside without stretch.

The phasic therapeutic rehabilitation has been showed in Table 3 [12-14]. Figure 1 shows the timeline and proceeding during the rehabilitation and Figure 2 shows the area of tenderness and Figure 3 shows the application of KT. Table 4 shows outcome measures.

**Table 3:** Phasic therapeutic rehabilitation. NA: not applicable; TENS: transcutaneous electrical nerve stimulation; Hz: hertz; reps: repetition; AP: anteroposterior; KT, kinesiology taping

Phase	Goal	Intervention	Dosage
Phase 1 (0-2 weeks)	Patient education	Educating about avoiding faulty running patterns. Suggestions about footwear modifications and proper running and surface	NA
	Recommendation	To rest and stop running	For at least 3 days
	To reduce pain	Cryotherapy	10 min
		TENS	10 min, pulse rate 150 Hz, pulse width 150 μs
		Muscle energy technique	10 reps for 10 sec hold with 3 sets
	To improve joint mobility	Talocrural joint distraction	3 reps of 15-20 secs
		Maitland mobilization for talocrural joint AP glides	Grade 3-4 for 3 reps × 2 min
		Lateral subtalar glide for subtalar joint	
	To facilitate the foot invertors and dorsiflexors	KT	Alternate day
Phase 2 (weeks 2-4)	To improve flexibility	Stretching for plantar flexors	10 reps × 3 sets with 30 sec hold
	Mild impact activities	Initiation of treadmill walking	0.8m/h for 10 min
	Initiate walking	Alternating 1 min normal and 1 min fast	30 min daily
		Progression: alternate walking for 1 min and jogging for 2 min	
		He was advised to concentrate on the heel-to-toe running pattern and to shift body weight forward during running.	
		After a few days, the patient ran easily for 5 min and walked for 1 min	
		Alternate walking for 1 min and jogging for 3 min	
	To enhance neuromuscular control and balance	Static and dynamic balance training on a wobble board progressing with eyes open and closed	20 mins
	To improve strength	Progressive resistance exercises with theraband for dorsiflexors and invertors	10 reps × 3 sets
Phase 3 (week 5-6)	Strength progression	Weighted calf raises, squats, lunges	10 reps × 3 sets
	Running progression	Run for 5 mins and walk for 1 min	30 mins
		Run for 10 mins and walk for 1 min	
		Run for 20 mins	
		Run 30 mins	

**Table 4:** Outcome measures used during the rehabilitation. NPRS: numerical pain rating scale; LEFS: lower extremity function scale; MTSS: medial tibial stress syndrome

Outcome measure	Pre-treatment	Post-treatment
NPRS	8/10	0/10
LEFS	32	100
MTSS score	9/10	0/10

## Discussion

MTSS is a common overuse injury in athletes and military personnel, characterized by pain along the inner side of the shinbone. The condition often results from repetitive weight-bearing activities and can progress from small injuries to more serious stress fractures. Studies show that KT can help manage MTSS by controlling navicular drop and reducing pressure shifts in the foot during running. KT provides better muscle support and maintains range of motion compared to rigid taping, making it a viable option for daily activities [15]. Radiological imaging should be used only when there is suspicion of other conditions, such as tibial stress fractures or osteosarcoma, rather than as a routine diagnostic tool for MTSS [16]. MTSS primarily follows a conservative approach, focusing on rest and adjusting activities to reduce repetitive, weight-bearing exercises. The length of rest needed for symptoms to improve differs from person to person, as there are no set recommendations. Other treatments with limited evidence of effectiveness are iontophoresis, phonophoresis, ice massage, ultrasound therapy, periosteal pecking, and extracorporeal shockwave therapy. On the other hand, treatments like low-energy laser therapy, lower leg braces, and compression stockings have not shown to be beneficial [17].

Griebert et al. found that utilizing anti-pronation KT led to a decrease in pressure on the medial side of the foot among individuals with MTSS. Their research delved into the effects of KT on biomechanical alterations and alleviation of symptoms in participants suffering from shin splints [18]. Naderi et al. found that arch-support foot orthoses did not produce a significant change in foot pressure distribution across different phases when compared to individuals who do not have MTSS. The results of their research indicate that these orthoses are successful in addressing the medial and lateral shifts of foot pressures during specific phases in MTSS. This suggests that arch-support orthoses could be a valuable intervention for correcting abnormal foot pressure distribution patterns while running, which may help in managing and avoiding MTSS in casual runners [8]. There is a lack of comprehensive research on the effectiveness of KT in treating MTSS. However, it appears that KT is effective in managing navicular drop and preventing the medial shift of the center of pressure during foot pronation deceleration [19]. The effectiveness of kinesiology tape (KT) in treating shin splints has not been extensively researched. However, KT appears to be effective in managing navicular drop and preventing the medial displacement of the center of pressure, while also reducing foot pronation [20].

The approach taken to manage MTSS in this novice runner yielded positive outcomes, in accordance with contemporary research findings. A notable decrease in pain was accomplished through a combination of rest, supportive aids, and focused physical therapy techniques. These methods are in line with current studies that underscore the necessity of rest and a gradual reintroduction to activity for the effective management of MTSS. The patient's successful and gradual reintegration into running, free from symptom recurrence, underscores the efficacy of a well-structured, progressive loading regimen, as corroborated by existing literature.

## Conclusions

This case report demonstrates that a comprehensive and individualized physical therapy approach can successfully manage MTSS in runners. The patient's favorable outcome, characterized by a significant decrease in pain, improved functional capacity, and a return to running without symptoms, underscores the importance of conservative strategies such as activity modification, rest, and targeted interventions like manual therapy and KT. In this particular case, KT was especially effective in addressing biomechanical issues, thereby enhancing traditional physical therapy practices. The results suggest that when implemented correctly, these interventions can play a crucial role in both the treatment and prevention of MTSS. Nonetheless, it is essential to recognize the limitations of this study, including its single-case design and the lack of long-term follow-up data, which may restrict the applicability of the findings. The effectiveness of these techniques, particularly KT, warrants further investigation to better understand their mechanisms and long-term benefits. Accurate diagnosis through clinical assessment is critical, with imaging primarily used to rule out more serious conditions. Future studies aim to assess the specific interventions utilized in this case and explore predictive factors in running biomechanics to enhance MTSS management strategies. The differential diagnosis for MTSS encompassed various conditions, including stress fractures, compartment syndrome, and tendonitis. Each condition was evaluated based on clinical manifestations and diagnostic evaluations. Stress fractures were excluded following a clinical assessment and the patient's reaction to conservative management, as imaging is generally necessary for definitive diagnosis. Compartment syndrome was ruled out due to the lack of accompanying symptoms such as intense, ongoing pain, and sensory impairments. Tendonitis was taken into account but was distinguished based on the pain's location and the patient's response to targeted treatment strategies.
